# Bidirectional Modulation of Neuronal Cells Electrical and Mechanical Properties Through Pristine and Functionalized Graphene Substrates

**DOI:** 10.3389/fnins.2021.811348

**Published:** 2022-01-11

**Authors:** Francesca Zummo, Pietro Esposito, Huilei Hou, Cecilia Wetzl, Gemma Rius, Raphaela Tkatchenko, Anton Guimera, Philippe Godignon, Maurizio Prato, Elisabet Prats-Alfonso, Alejandro Criado, Denis Scaini

**Affiliations:** ^1^Neuroscience Area, International School for Advanced Studies (SISSA), Trieste, Italy; ^2^Center for Cooperative Research in Biomaterials (CIC biomaGUNE), Basque Research and Technology Alliance (BRTA), San Sebastián, Spain; ^3^Institut de Microelectrònica de Barcelona, IMB-CNM (CSIC), Esfera UAB, Bellaterra, Spain; ^4^Centro de Investigación Biomédica en Red en Bioingeniería, Biomateriales y Nanomedicina (CIBER-BBN), Madrid, Spain; ^5^Department of Chemical and Pharmaceutical Sciences, University of Trieste, Trieste, Italy; ^6^Basque Foundation for Science (IKERBASQUE), Bilbao, Spain; ^7^Centro de Investigacións Científicas Avanzadas (CICA), Universidade da Coruña, A Coruña, Spain; ^8^Nanomedicine Research Laboratory, Department of Medicine, Imperial College London, Hammersmith Hospital, London, United Kingdom

**Keywords:** hippocampal neurons, graphene, chemical functionalization, synaptic activity, cell stiffness

## Abstract

In recent years, the quest for surface modifications to promote neuronal cell interfacing and modulation has risen. This course is justified by the requirements of emerging technological and medical approaches attempting to effectively interact with central nervous system cells, as in the case of brain-machine interfaces or neuroprosthetic. In that regard, the remarkable cytocompatibility and ease of chemical functionalization characterizing surface-immobilized graphene-based nanomaterials (GBNs) make them increasingly appealing for these purposes. Here, we compared the (morpho)mechanical and functional adaptation of rat primary hippocampal neurons when interfaced with surfaces covered with pristine single-layer graphene (pSLG) and phenylacetic acid-functionalized single-layer graphene (fSLG). Our results confirmed the intrinsic ability of glass-supported single-layer graphene to boost neuronal activity highlighting, conversely, the downturn inducible by the surface insertion of phenylacetic acid moieties. fSLG-interfaced neurons showed a significant reduction in spontaneous postsynaptic currents (PSCs), coupled to reduced cell stiffness and altered focal adhesion organization compared to control samples. Overall, we have here demonstrated that graphene substrates, both pristine and functionalized, could be alternatively used to intrinsically promote or depress neuronal activity in primary hippocampal cultures.

## Introduction

Graphene is an atomically-thin carbon-based nanomaterial currently exploited in many research fields (Randviir et al., 2014), as well as industrial sectors (Zhou et al., 2011; Torrisi et al., 2012), including advanced biomedical applications (Ryoo et al., 2010; Nayak et al., 2011; Zhang et al., 2011; Reina et al., 2017). In regard to the latter application, graphene unusual physicochemical properties, such as its high carrier mobility, optical transparency and ease of chemical functionalization, open to novel approaches in the design and fabrication of advanced neuronal tools as, for example, implanted brain sensors, smart stimulation electrodes or neuroprosthetic devices (Lu et al., 2016; Shin et al., 2016; Franceschi Biagioni et al., 2021). In this framework, the possibility to layer graphene on virtually every surface and its ease of chemical functionalization (Xu et al., 2016), drastically expands material capabilities making it possible, for example, to design devices able to tune cellular adhesion (Jeong et al., 2016), reduce inflammatory responses (Zhou et al., 2016), or introduce chemical moieties (Karki et al., 2020). Among all chemical functionalization able to alter surface physicochemical properties, the amino (-NH_2_) and carboxyl groups (-COOH) have demonstrated to induce cell adhesion and promote cell growth (Keselowsky et al., 2004; Ren et al., 2009; Deng et al., 2015), presumably through an improved cell wettability associated with surface charging and protein adsorption (Arima and Iwata, 2007).

Unfortunately, despite hopes raised by recent development in graphene applications, the understanding of its functional interactions with brain tissue when chemically functionalized is still limited, particularly concerning the synergic effect of chemical moieties and graphene effect on neuronal excitability. Indeed, glass-supported single-layer graphene has demonstrated an intrinsic ability to induce a boost in the electrical activity of interfaced neuronal cells (Tang et al., 2013; Kitko et al., 2018; Pampaloni et al., 2018a). This effect has been reconducted to the ability of graphene to perturb the distribution of extracellular ions at the interface with neurons giving rise to altered ion currents, associated with increased firing rate. Apparently, the graphene–ion interactions responsible for the effect occur when single-layer graphene is laid on electrically insulating substrates but vanish on electrically conductive ones (Pampaloni et al., 2018b). Other studies reconducted graphene-induced neurotransmission potentiation to an alteration in cholesterol homeostasis impacting the number, release probability, and recycling rate of synaptic vesicles (Kitko et al., 2018). A common aspect of all these studies is the validation of the possibility to potentiate the spontaneous electrical activity of a neuronal network simply by facing it with graphene. This remarkable result allows the possible use of the material for neuromodulation. However, the limitation to induce only network excitation limits the effectiveness of neuromodulation due to the lack of the ability to downregulate the electrical activity.

In the present study, we attempted to respond to this need by culturing primary neurons from rat hippocampus above glass-supported pristine single-layer graphene (pSLG) and phenylacetic acid functionalized graphene (fSLG), comparing their properties to those of cells plated on bare glass controls. The carboxyl group conveyed by the phenylacetic acid surface insertion, in particular, has already been demonstrated to allow effective cell attachment and development with a very mild effect on cell vitality and functionality (Lee et al., 2006; Ren et al., 2009). Indeed, this chemical group represents a valuable validation molecule to investigate the impact of chemical functionalization on graphene. We aimed at disclosing the synergic effect of graphene and chemical groups on neuronal network development focusing, in particular, on its impact on neuronal cell electrical and biomechanical properties. To pursue the investigation, we directly plated cells on graphene substrates and evaluated network morphology and composition by immunofluorescence, electrical activity by patch-clamping recordings, and cell stiffness by Atomic Force Microscopy (AFM) force-spectroscopy experiments.

## Materials and Methods

### Preparation of Single-Layer Graphene Substrates

Single-layer graphene (SLG) was grown by chemical vapor deposition (CVD) on flat Cu foils and subsequently transferred onto supporting glass substrates. In brief, graphene was produced using a Black MagicPro 4” reactor on a 25 μm thick copper foil (99.8% metal basis, Alfa Aesar, United Kingdom). Before the CVD process, copper foils were cut in 6 cm^2^ × 5 cm^2^ samples, cleaned in acetic acid and acetone, and finally rinsed in isopropyl alcohol (IPA). The two-steps growth process consisted of a 10 min Cu surface reduction at 1,000°C by flowing 400 standard cubic centimeters per minute (sccm) of H_2_, 600 sccm of Ar and 150 sccm of N_2,_ followed by a 20 min graphene growth at 970°C using methane as carbon precursor (10/100 sccm CH_4_/ H_2_ ratio). A 700 nm thick sacrificial layer of polymethyl methacrylate (PMMA) was spin-coated above the graphene (7% 950k MW dissolved in anisole, Micro Resist Technology GmbH, DE). After a soft backing, specimens of about 10 mm^2^ × 10 mm^2^ in edge size were cut out. The graphene layer was separated from copper using an electrochemical delamination process (De La Rosa et al., 2013) and, after washing in deionized water, transferred onto supporting glass coverslips (12 mm^2^ × 24 mm^2^, No. 1, 0.13–0.16 mm in thickness, ORSAtec GmbH, DE). Before the transfer procedure, supporting substrates were ultrasonicated in acetone and isopropanol (IPA) to assure the required cleanness and activated with a UV-ozone treatment (UVO, 5 min). After transfer, samples were let to dry and baked at 180°C for 2 min. The PMMA sacrificial layer was removed by immersing samples in acetone and then in IPA, 30 min in each solvent. pSLG substrates were used as transferred while, in fSLG, phenyl acetic acid moieties were immobilized on the surface. The functionalization was accomplished by placing some of the pSLG substrates in a glass beaker with distilled water (10 mL). Then, a distilled water solution of 4-(carboxymethyl)phenyl diazonium tetrafluoroborate (10 mg, 20 mM final concentration, synthesized following a published diazotization procedure (Franco et al., 2020) was slowly added with a syringe pump (2.5 mL/h) for 1 h at room temperature (RT). Substrates were subsequently removed from the solution and cleaned by immersion (3 times) in distilled water and dried over a stream of N_2_.

The graphene materials were characterized by Raman spectroscopy and AFM. To evaluate the quality of graphene obtained and to quantify the functionalization degree, the ratio between the D (∼1,345 cm^–1^) and G (∼1,589 cm^–1^) Raman band intensities of graphene (I_*D*_/I_*G*_) were employed (Bottari et al., 2017). Raman spectra were recorded with a Renishaw inVia™ Raman spectrometer equipped with a green laser (λ = 532 nm) and plotted after baseline correction using the Wire 4.4 software. An average defect density (*nD*) of 4.38⋅10^–4^ nm^–2^ was obtained for fSLG samples. Defects were interpreted as changes in the graphene lattice C hybridization from sp^2^ to sp^3^ induced by the covalent modification (Cançado et al., 2011). Topographical changes of the surface of graphene were evaluated by performing AFM analysis (see Atomic Force Microscopy section).

### Cell Cultures Preparation

Dissociated hippocampal cells were derived from P0–P3 old rats as described in the literature (Lovat et al., 2005; Cellot et al., 2011; Pampaloni et al., 2018b; Rago et al., 2019). Briefly, rat hippocampi were isolated and digested in trypsin and deoxyribonuclease (6,000 and 1,560 U/mL, respectively, both from Sigma Aldrich). After digestion, the cell suspension was centrifuged (800 rpm for 5 min), and the pellet was collected and resuspended in a fresh culture medium composed of Neurobasal-A medium (Thermo-Fischer) supplemented by B27 (2%, Gibco) and Glutamax^®^ (10 mM, Gibco). Cells were plated on polyornithine-coated glass coverslips controls and on uncoated pSLG and fSLG substrates at a nominal concentration of 10^6^ cells/mL (300 μL for 45 min). Before cell plating, all substrates were sterilized for 30 min under UV light. Cultures were maintained in incubation conditions (37 °C, 5% CO_2_, 95% RU) in 35 mm petri dishes containing about 2 mL of culture medium. All the experiments were performed after 8–10 days *in vitro* (DIV).

All animal procedures were conducted following the indications of the National Institutes of Health and international and institutional standards for the care and use of animals in research. All experiments were performed in agreement with the Italian law (decree 26/14) and the European Union (EU) guidelines (2007/526/CE and 2010/63/UE) and were approved by the local authority veterinary service and by our institutional (SISSA-ISAS) ethical committee. All efforts were made to minimize animal suffering and reduce the number of animals necessary to accomplish our studies.

### Immunofluorescence

Cells were fixed with 4% paraformaldehyde (PFA) in phosphate buffered saline (PBS) for 20 min. Subsequently, samples were incubated for 1 h at RT in a PBS blocking solution containing 5% fetal bovine serum (FBS) and 0.03% Triton X-100 to permeabilize cell membranes. After rinsing in PBS, primary antibodies were added for 1 h at RT and, after a PBS washing, secondary antibodies were added for 45 min in dark conditions (Cellot et al., 2016; Rago et al., 2019). As primary antibodies were used: mouse monoclonal anti-NeuN (MAB377, Sigma-Aldrich, 1:500 dilution), rabbit polyclonal anti-GFAP (HPA056030, Sigma-Aldrich, 1:500 dilution), rabbit polyclonal anti-β-tubulin III (T2200, Sigma-Aldrich, 1:250 dilution), and mouse monoclonal anti-GFAP (G3893, Sigma-Aldrich, 1:250 dilution). As secondary antibodies were instead used: AlexaFluor^®^ 594 goat anti-mouse (A11032, Sigma-Aldrich, dilution 1:500) and anti-rabbit (A11037, Life Technologies, dilution 1:500), and AlexaFluor^®^ 488 goat anti-mouse (A11029, Life Technologies, dilution 1:500) and anti-rabbit (A11034, Sigma-Aldrich, dilution 1:500). Unspecific cell-nuclei staining was performed using 4′,6-diamidino-2-phenylindole (DAPI, D1306, Thermo Fisher, dilution 1:200). Samples were mounted on standard microscope glass slides (Fisher Scientific) using Fluoromount-G™ Mounting Medium (Thermofisher). NeuN and DAPI stainings were used to compute neuronal *vs* non-neuronal cell densities. Specifically, images 636 μm × 636 μm (1024 pixels × 1024 pixels) were acquired with an epifluorescence microscope (DM6000, Leica Microsystems) using 20× objective (0.5 N.A). Three visual fields per condition were randomly collected (2 samples per condition per session from three independent cell-culture series). The offline analysis was conducted semi-automatically in Fiji (Schindelin et al., 2012), using the cell-count plugin developed by Grishagin (2015). Cell shape and network morphology were instead highlighted by immunolabeling neurons against β-tubulin III, and astrocytes against the glial fibrillary acidic protein (GFAP). Images were acquired using a confocal microscope (Nikon eclipse C1 equipped with Ar/Kr and He/Ne lasers) using a 60× oil-immersion objective (Plan Apo, 1.40 NA, Nikon Corporation). Images were recorded at 212 μm × 212 μm (1024 pixels × 1024 pixels) collecting about 14 focal planes per field (600 nm Z-stacks step-length).

### Electrophysiology

All patch-clamp recordings were performed in voltage clamp mode in whole-cell configuration at RT. The glass pipettes, thermally pulled to achieve a final resistance of 4–8 MΩ, were filled with an intracellular solution composed of 120 mM K-gluconate, 20 mM KCl, 10 mM HEPES, 10 mM EGTA, 2 mM MgCl_2_ and 2 mM Na_2_ATP (pH 7.3; 300 mOsm in osmolarity, all compounds from Sigma Aldrich) and immersed in an extracellular recording solution composed by 150 mM NaCl, 4 mM KCl, 1 mM MgCl_2_, 2 mM CaCl_2_, 1 mM MgCl_2_, 10 mM HEPES and 10 mM glucose (pH 7.4; about 320 mOsm in osmolarity, all compounds from Sigma Aldrich). Data were collected using a Patch Clamp EPC 7 patch amplifier (HEKA Electronic, United States) and digitized using a Digidata 1322A (Molecular Devices LLC, United States) at 10 kHz sampling frequency using the pClamp 8.2 acquisition software (Molecular Devices LLC, United States). Cell membrane passive properties (input resistance R_m_, and membrane capacitance C_m_), were measured by averaging the cell response to 80 voltage steps (-5 mV, 10 ms in duration) in terms of currents through the Clampfit software (pClamp 10.3, Molecular Devices LLC, United States). Uncompensated series resistance was less than 11 MΩ. Spontaneous post-synaptic currents (PSCs) were recorded by clamping the membrane voltage at -56 mV holding potential (value not corrected for the liquid junction potential, calculated to be equal to -14 mV). Miniature post-synaptic currents (mPSCs) were instead recorded in the presence of 1 μM Tetrodotoxin (TTX, Latoxan), a specific blocker of fast-activating voltage-gated Na^+^ channels. The analysis of synaptic events was performed offline using the AxoGraph neuronal event detection software (version 1.7.0, Axon Scientific, United States). Specifically, for each recording all events detected were averaged and the peak amplitude and kinetic properties of the resulting mean current were measured.

### Atomic Force Microscopy

Glass control, pSLG and fSLG substrate surfaces were characterized by AFM. A commercial Solver PRO AFM instrument (NT-MDT Co., RU) was used in no-contact mode in air (normal pressure and RT). Both topographic and phase signal images were acquired using silicon AFM tips (Etalon^®^ HA-NC rectangular cantilevers, spring constant 12 nN/nm, resonant frequency 235 kHz, probe tip radius 10 nm, NT-MDT Co., RU). Images of 2.5 × 2.5 μm^2^ (512 × 512 pixels^2^) were acquired at 1 lines/second scan speed. The open-source SPM analysis software Gwyddion (Nečas and Klapetek, 2012) was used to process and analyze all AFM images. Root mean square line-roughness (R_*RMS*_) was defined internally to the software and corresponds to the average of the height deviations from the mean line of the selected profile.

The stiffness of neuronal cells interfaced to glass, pSLG and fSLG substrates was evaluated through AFM force-indentation experiments. Measurements were carried out at RT in liquid environment (PBS). Stiffness assessment was conducted taking advantage of the cell-mechanics analysis capability integrated into the AFM tool (JPK NanoWizard^®^ 3, Bruker Nano Surfaces, United States). Experiments were performed using tip-less cantilevers with a nominal elastic constant of 0.03 nN/nm and a resonance frequency of 10 kHz (CSG11-B/tipless, NT-MDT Co., Russia) on which apex a glass bead (8.0 ± 0.4 μm in diameter, No. 9008, Duke Standards™, Fremont, CA, United States) was glued using a UV-curable glue (Norland Optical Adhesive 61, Norland Products, Inc., United States). Cantilevers’ effective elastic constants were determined by the software-integrated thermal method (Lévy and Maaloum, 2002). Neuronal cell stiffness has been evaluated after having fixed cells in 4% PFA in PBS and made them visible by NeuN staining. Although cell fixation is known to increase cell stiffness, it has been shown that it is possible to use these values to perform a relative comparison of cell mechanical adaptation on the three different substrates (Jiang et al., 2011; Grimm et al., 2014; Ulloa et al., 2021). Indentation was conducted by manually placing the cantilever tip above the NeuN-stained cell soma. This strategy ensures high measurement reproducibility. Measurements were performed at a constant speed of 2.5 μm/s and triggered to a maximum applied force of 5 nN and never exceeded an indentation depth of 250 nm, representing 5% of the average measured cell height (data not shown). Such maximum indentation avoided any contribution from the underneath substrate to the measured cell stiffness minimizing, at the same time, its susceptibility to cell nucleus stiffness. Cell stiffnesses were determined using the JPKSPM Data Processing^®^ software by fitting the obtained force-indentation curves with the integrated Hertzian model for a spherical indenter (Sneddon, 1965). Averaging values were computed from measurements performed on about 200 cells per condition from 3 independent experiments. Neuronal stiffness was reported in terms of Young’s Modulus (E) and expressed in kPa.

### Total Internal Reflection Fluorescence

Total internal reflection fluorescence (TIRF) experiments were performed to evaluate the number and distribution of focal adhesions (FAs) in hippocampal cultures grown above glass, pSLG and fSLG substrates. Cellular samples were fixed with 4% PFA for 30 min at RT. After fixation, samples were incubated for 1 h in a PBS blocking solution containing 5% fetal bovine serum (FBS) and 0.03% Triton X-100 to block non-specific sites and permeabilized cells. Subsequently, they were incubated for 1.5 h at RT with a monoclonal mouse anti-vinculin antibody (V9131, Sigma-Aldrich, 1:400 dilution in blocking solution without Triton) and a rabbit polyclonal anti-β-tubulin III (T2200, Sigma-Aldrich, 1:250 dilution in blocking solution without Triton). Then, samples, washed with PBS, were incubated for 1 h with a goat anti-mouse secondary antibody coupled to Alexa Fluor^®^ 488 (A11029, Life Technologies, 1:500 dilution) and Alexa Fluor^®^ 594 goat anti-rabbit secondary antibody (A11037, Life Technologies, 1:500 dilution), diluted in blocking solution without Triton. Samples were mounted on standard microscope glass slides (7525M, J. Melvin Freed, United States) using a liquid mounting medium (Fluoromont-G™, ThermoFisher), and then visualized using an inverted epi-fluorescence microscope equipped with a TIRF module (Nikon Eclipse-TiU, Nikon Corporation). Images were acquired in total reflection condition using a high aperture oil-immersion objective (CFI Apochromat TIRF 100 × C Oil 1.49 N.A., Nikon Corporation) and a 488 nm laser (OBIS 488 LS; Coherent, Inc., Santa Clara, CA, United States) at a power sufficient to avoiding photo-bleaching. In our conditions, the penetration depth of the evanescent wave was about 150 nm. Samples were visualized using a CCD camera (DS-Qi1, Nikon Corporation) acquiring for every field of view a TIRF image of the vinculin staining and a conventional fluorescence image of β-tubulin III positive regions (426 μm^2^ × 340 μm^2^, 1280 × 1024 pixels^2^). FAs were quantified as vinculin-positive puncta exploiting a procedure developed within the Wolfram Mathematica software suite (version 12.3.0, Wolfram Research, Inc., United States). Briefly, vinculin signal was analyzed exclusively in β-tubulin positive regions. After image binarization, puncta were highlighted using a morphological-matching procedure based on disks of increasing radius (from 300 nm to 3 μm) and segmented. For every condition, the area of every detected punctum was computed and averaged. FAs density was evaluated as the ratio between the total number of puncta and the area of β-tubulin positive regions.

### Statistical Analysis

All the described experiments were repeated at least three times using cell cultures from independent experimental sessions. All statistical analysis was performed using the Prism software (version 6.0, GraphPad). Data distribution was evaluated by the Shapiro Wilk test of normality and, based on the result, a bar chart showing mean ± standard deviation (SD) or a box plot representation was chosen to graphically represent data. Box plots are plotted as median with boxes spanning from the 25th (1st quartile, Q1) to the 75th (3rd quartile, Q3) percentiles, with whiskers representing the 5th and 95th percentiles. Statistics between two independent samples were performed with t-test when the distribution was normal. For parametric data Student’s *t*-test was used to compare two independent conditions, while comparisons between more than two conditions were performed performing a one-way ANOVA analysis followed by Tukey’s multiple comparison post-hoc test. For non-parametric data, Mann Whitney for two groups comparison test or Kruskal-Wallis followed by Dunn’s multiple comparison test were used. For the sake of clarity, all the values reported in the main text are expressed as mean ± SD. Statistical significance was determined at P < 0.05, unless otherwise indicated. Significance was graphically indicated as follows: *P < 0.05, ^**^P < 0.01, ^***^P < 0.001.

## Results

### Materials Fabrication and Characterization

**FIGURE 1:**
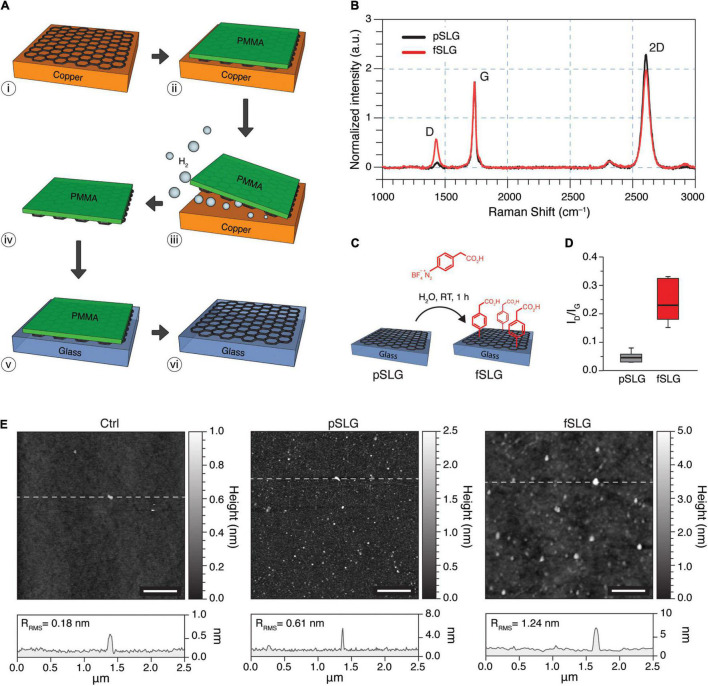
Fabrication and characterization of pSLG and fSLG. **(A)** Scheme of the graphene transfer process from copper to glass exploiting an electrochemical delamination procedure: (i) CVD-grown single-layer graphene on copper; (ii) deposition of a supportive PMMA film; (iii) electrochemical delamination; (iv) free-standing graphene/PMMA layers; (v) transfer on glass; (vi) dissolution of the sacrificial PMMA layer. **(B)** Average of 20 randomly-acquired Raman spectra of a graphene sample before the chemical functionalization (pSLG, in black) and after (fSLG, in red) exhibiting the typical bands of CVD graphene in both conditions (ΔI_*D*_/I_*G*_ of about 0.29). It is worth noting that peaks’ shape was impacted by the sporadic presence of double layer nucleation sites. **(C)** Schematic of the diazonium coupling onto pSLG by using 4-(carboxymethyl)benzene diazonium tetrafluoroborate to obtain fSLG. **(D)** Box plot of the I_*D*_/I_*G*_ ratio for pSLG and fSLG. The plot showed a significant change after functionalization reaction, which confirms the covalent modification of graphene. **(E)** AFM topographic images of glass (left), pSLG (middle), and fSLG (right) surfaces before cell plating. Below, the representative topographic profiles relative to the highlighted lines in the images are shown.

Single-layer graphene was grown on flat Cu foils, separated from copper using an electrochemical delamination process, and subsequently transferred onto supporting glass substrates (Figure 1A). The quality of CVD-grown pSLG and the effectiveness of chemical functionalization of fSLG were assessed by Raman spectroscopy analysis (Figure 1B). Specifically, the distinctive graphene Raman spectrum is characterized by the signature bands so-called D, G and 2D (Reina et al., 2009; Dong et al., 2011; Nguyen et al., 2013). The D peak, localizes at about 1,345 cm^–1^, is more prominent when chemical or structural non-idealities are present, such as impurities or defects, but it can also be correlated with covalent chemical functionalization (Criado et al., 2015). The G peak, placed at about 1,589 cm^–1^, is characteristic of sp^2^ hybridization showing the structural order and purity in all graphitic materials whereas, the 2D peak, sited at about 2,689 cm^–1^, is characteristic of the atomically-thick 2D nature of graphene films. For instance, peaks’ characteristics analysis allows the discrimination between single- versus multi-layered graphene, as reported in the literature (Ferrari et al., 2006; Faugeras et al., 2009; Malard et al., 2009). Precisely, it is well-known that the ratio of the intensity of the 2D and G bands (I_2D_/I_*G*_) is indicative of the number of graphene layers. A 2 < I_2D_/I_*G*_< 3 ratio is indicative of monolayer graphene, 1 < I_2D_/I_*G*_< 2 of bilayer graphene, while I_2D_/I_*G*_< 1 is associated with the multilayer one. In addition, the presence of single-layer graphene can also be evinced by analyzing the shape of the 2D peak, where a full width at half maximum (FWHM_2D_) value of about 38 cm^–1^ is indicative of a single-layer material, even in the presence of I_2D_/I_*G*_ value falling between 1 and 2. Based on measured I_2D_/I_*G*_ and FWMH_2D_ values, we can state that the graphene films grown on the Cu foil at 970°C were mainly a monolayer with a contingent distribution of double-layer nucleation sites.

Pristine SLG was chemically modified through a radical reaction employing 4-(carboxymethyl)phenyl diazonium tetrafluoroborate (Figure 1C). The diazonium salt reacts with graphene through a single electron transfer (SET) mechanism, resulting in a change in the hybridization of carbon atoms within the graphene lattice from sp^2^ to sp^3^ (Paulus et al., 2013). Chemically modified SLG samples were also characterized by Raman spectroscopy (Figure 1B, red line) and AFM (Figure 1E) to confirm and evaluate the chemical modification. Based on Raman scattering, the successful chemical modification was confirmed by a significant increment in the I_*D*_/I_*G*_ ratio (Figure 1D). The increased intensity of the D peak evidenced the change in the hybridization of carbon atoms, as defects in the structure of graphene, which would be purely sp^2^ (Figure 1B).

The quantification of the degree of chemical functionalization was carried out by evaluating the defect density (*nD*) (Cançado et al., 2011) using the following relation:


$$n\,D\,\left(c\,m^{-2}\right)=\frac{\left(1.8\pm 0.5\right)\times 10^{22}}{\lambda_{L}^{4}}\cdot \left(\frac{I_{D}}{I_{G}}\right)$$

where λ_*L*_ is the excitation wavelength. It is worth noting that this equation is valid for Raman data obtained from graphene samples with distances between defects ≥10 nm. Thus, the fSLG samples showed a moderate average *nD* = 4.38⋅10^–4^ nm^–2^ (Supplementary Table 1).

The morphology of pristine and phenylacetic acid functionalized SLG films were characterized by AFM and compared to bare glass coverslips control substrates (Figure 1E). Line profile analysis revealed an increase in surface roughness compared to glass (Ctrl), for both pSLG and fSLG (Figure 1E, bottom; R_*RMS*_ = 0.18 nm for Ctrl, R_*RMS*_ = 0.61 nm for pSLG, and R_*RMS*_ = 1.24 nm for fSLG), as apparent in the three topographical reconstructions. The qualitative change in SLG surface morphology after the chemical modification, quantified in terms of an increased roughness, can be correlated to the well-known oligomers derived from the generated phenyl radicals (Hossain et al., 2010).

### Graphene Substrates Support the Development of Hippocampal Neuronal Networks

Even though several reports have already demonstrated the successful development of hippocampal cell cultures interfaced with graphene substrates (Tang et al., 2013; Fabbro et al., 2016; Kitko et al., 2018; Pampaloni et al., 2018a; Rauti et al., 2020b), this aspect has not been evaluated on chemically functionalized graphene yet. To explore the potential effects of graphene and its chemical functionalization on cell development and network composition, we cultured primary hippocampal neurons on both pSLG and fSLG supports. Neurons plated directly on glass coverslips were used as control cultures (Lovat et al., 2005; Cellot et al., 2009; Pampaloni et al., 2018b; Rago et al., 2019).

**FIGURE 2:**
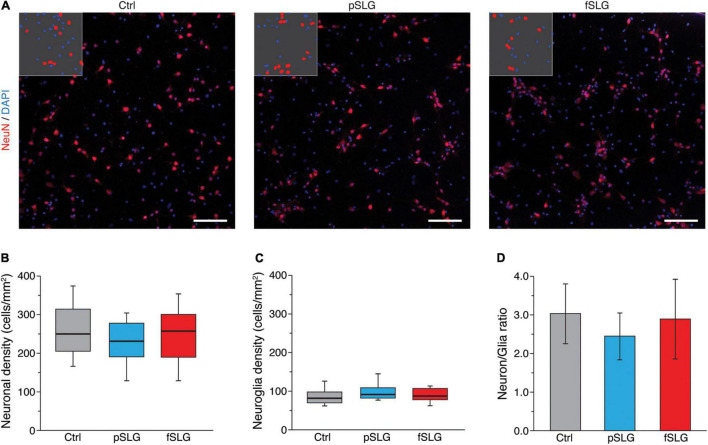
Graphene-based substrates do not affect cell density. **(A)** Representative fluorescence images of hippocampal cells on glass control (left), pSLG (middle) and fSLG (right). Cells were stained for NeuN (red) and DAPI (blue). Insets show representative portions of the images after the binarization and segmentation procedure highlighting DAPI-positive regions and NeuN-positive ones. Scale bars: 100 μm. **(B)** Box plot of neuronal cells densities showing no significant difference across the three conditions (*p* > 0.05). **(C)** Box plot of glial cells density. No statistically significant differences were found between experimental groups (*p* > 0.05). **(D)** Bars plot highlighting the consistency of the neuronal/glial cells density ratio across the three conditions (*p* > 0.05).

Both graphene substrates, pSLG and fSLG, were able to sustain a similar growth and development of hippocampal cells with respect to the glass control condition. We qualitatively investigated the network size and phenotypical composition evaluating the density of neuronal and non-neuronal cells. Specifically, we marked the nuclei of all the cells of our cultures using 4′,6-diamidino-2-phenylindole (DAPI) and specifically highlighted neurons by immunolabelling them against NeuN, a protein localized in nuclei and perinuclear cytoplasm of most of the neurons in the central nervous system of mammals (Sarnat et al., 1998; Figure 2A). An automatic procedure was used to binarize images and classify cells as neuronal or non-neuronal (Figure 2A, inlets). The non-neuronal constituent of primary hippocampal cultures is neuroglia (Jäkel and Dimou, 2017) which, in our experimental conditions, is mainly composed by astrocytes (Lovat et al., 2005; Cellot et al., 2016; Rauti et al., 2020a). No significant differences were detected across all three substrates in terms of neuronal density (Figure 2B) or neuroglia density (Figure 2C). Similarly, the ratio between neuronal and glial cells appeared to be similar across all three conditions (Figure 2D).

### Graphene Substrates Alter Neuronal Electrical Activity

**FIGURE 3:**
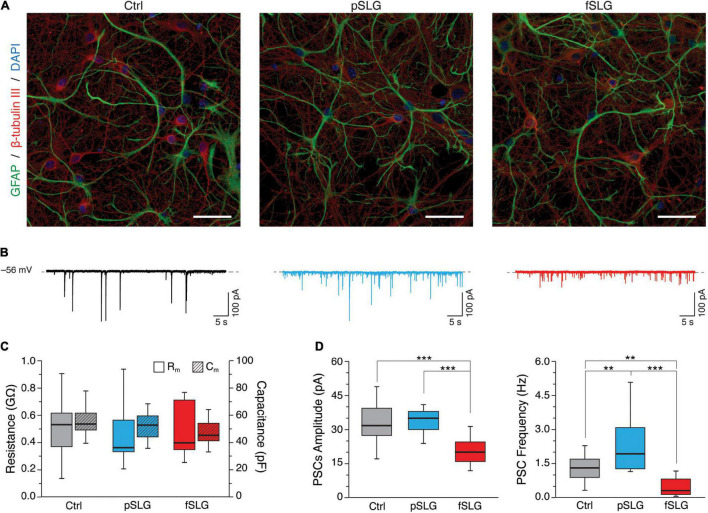
Graphene-based substrates modulate neuronal network activity. **(A)** Representative confocal micrographs of hippocampal cells grown on glass control, pSLG and fSLG. Neurons were labeled against β-tubulin III (red), astrocytes against GFAP (green) and nuclei with DAPI (blue). Scale bars: 40 μm. **(B)** Representative voltage-clamp current traces for controls (in black, on the left), pSLG (in blue, on the middle) and fSLG (in red, on the right). **(C)** Box plot summarizing neuronal cell membrane resistances (R_m_, plain boxes) and capacitances (R_c_, striped boxes) across the three supporting substrates. **(D)** Boxplot of PSCs amplitudes (left) and frequencies (right). A significant difference was detected only in fSLG PSCs amplitudes, while pSLG and fSLG oppositely modulated neuronal PSCs frequencies (up-regulating and down-regulating, respectively). Significance: **p < 0.01, ***p < 0.001.

To assess the impact of graphene substrates on the cellular morphology of the interfaced networks, we revealed by immunofluorescence the shape of neurons and astrocytes. For that purpose, we marked the neuronal cytoskeletal components β-tubulin III and the glial fibrillary acidic protein (GFAP), while cells nuclei were stained using DAPI (Figure 3A). Confocal micrographs show that cells adopt a healthy morphology without any apparent difference across the three conditions.

We further extended our investigation by performing a functional analysis of the spontaneous synaptic activity characterizing our neuronal networks interfaced to graphene and glass for 8–10 DIV. Voltage-clamp electrophysiological experiments were conducted by patching neurons within the three conditions and recording network spontaneous post-synaptic currents. Heterogeneous PSCs were detected as inward currents of variable amplitudes (Lovat et al., 2005) in all conditions, as shown by the three representative traces in Figure 3B.

Plasma membrane passive properties, measured for each patched neuron, provided further indications about cell viability and membrane integrity in cells developed above pSLG and fSLG substrates. We compared the results with glass controls and no significant differences were identified in terms of input resistance and membrane capacitance over the three experimental groups (Figure 3C; 519 ± 208 MΩ and 54.8 ± 8.5 pF for Ctrl, *n* = 23 cells; 438 ± 134 MΩ and 51.7 ± 9.6 pF for pSLG, *n* = 16; 493 ± 188 MΩ and 47.2 ± 9.1 pF for fSLG, *n* = 16).

The appearance of spontaneous post-synaptic events in all of our traces was both evidence of functional synaptic formation and an index of network efficacy (Lovat et al., 2005; Pampaloni et al., 2018a). Interestingly, the quantification of the amplitude and frequency of PSCs in pSLG and fSLG interfaced neurons revealed a different functional adaptation of these networks when compared to glass. Specifically, our data highlighted that while PSCs amplitudes of networks developed above glass and pSLG were almost identical, pSLG interfaced neuronal networks showed a significant enhancement in the frequency of synaptic currents with respect to controls (Figure 3D, gray and blue boxes; 32.62 ± 7.65 pA and 1.31 ± 0.56 Hz for glass controls, *n* = 23 cells; 33.76 ± 5.11 pA and 2.33 ± 1.24 Hz for pSLG, *n* = 16). In contrast, fSLG induced a significant reduction in both PSCs amplitude and frequency in interfaced networks compared with pSLG and glass (Figure 3D, red boxes; 21.74 ± 8.4 pA and 0.48 ± 0.40 Hz for fSLG, *n* = 16 cells).

**FIGURE 4:**
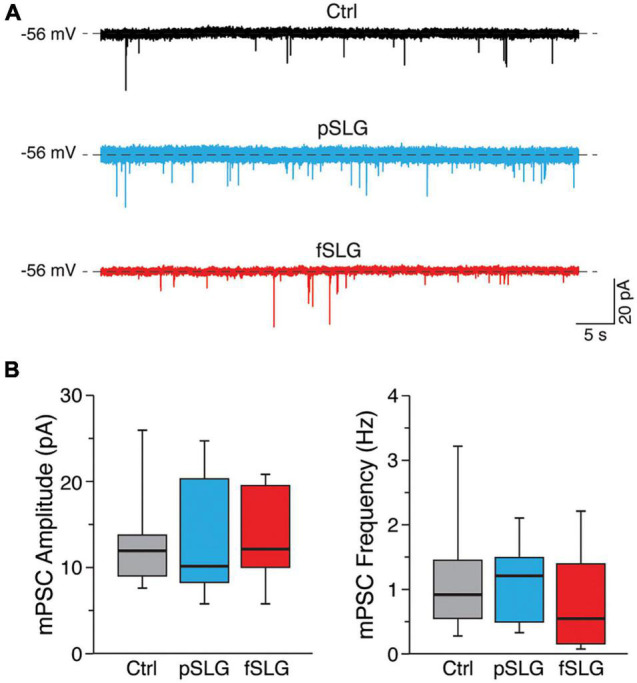
Graphene-based substrates do not alter the structural functionality of synapses at pre- and post-synaptic levels. **(A)** Representative traces of mPSCs for the three conditions under investigation. **(B)** Box plots of mPSCs amplitudes (left) and frequencies (right) measured from neuronal networks developed above glass, pSLG and fSLG substrates. No significant differences were detected across the three experimental groups regarding both mPSCs amplitudes nor frequencies (*p* > 0.05).

To move through this phenomenon, we tested if the changes in synaptic activity detected in our experiments could result from a structural reassembling at the synaptic level. We measured miniature postsynaptic currents (mPSCs) by application of TTX (Figure 4A). For each condition we evaluated the amplitude and frequency of mPSCs. The former gives an indication about the number of neurotransmitter receptors localized at the post-synaptic terminal, whereas the latter is mainly influenced by the pre-synaptic release probability and by the number of synaptic contacts (Raastad et al., 1992; Pampaloni et al., 2018a). Interestingly, no significant difference in amplitudes nor frequencies were detected across the three conditions (Figure 4B; 13.2 ± 6.2 pA and 1.1 ± 0.85 Hz for glass, *n* = 10 cells analyzed; 13.9 ± 7.2 pA and 1 ± 0.5 Hz for pSLG, *n* = 10; 15.2 ± 6.8 pA and 0.8 ± 0.7 Hz for fSLG, *n* = 10). Consequently, we can rule out that pristine or phenylacetic acid modified graphene substrates alter the structural functionality of synapses at pre- and post-synaptic level, excluding that such phenomenon stays behind the observed changes in network activity.

### Focal Adhesion Organization and Cell Stiffness

**FIGURE 5:**
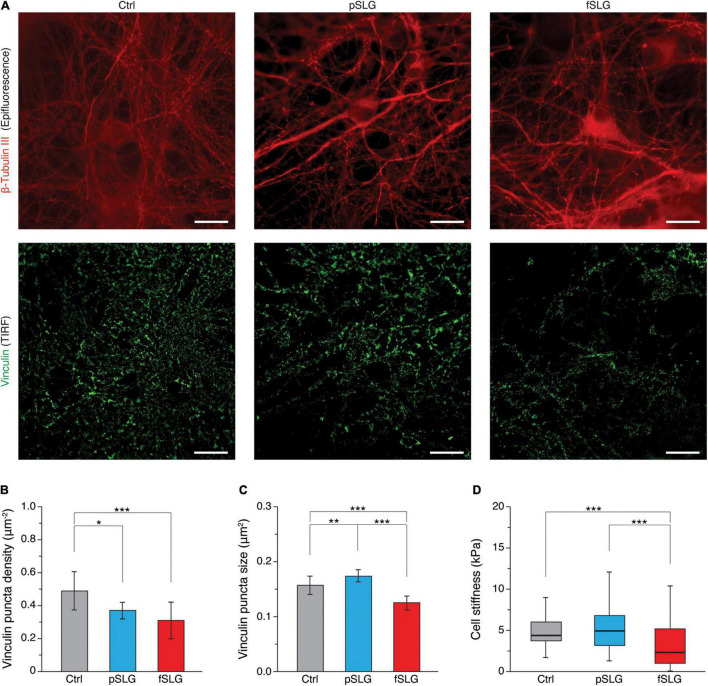
Focal adhesion distribution and cell stiffness. **(A)** TIRF-based investigation of hippocampal cells on glass control (left column), pSLG (central column) and fSLG (right column). Micrographs in the first row show cells stained against β-tubulin III (in red, acquired as an epi-fluorescence signal), those in the second row show cells labeled against vinculin (in green, acquired as a TIRF signal). Scale bars: 20 μm. **(B)** Bar plot summarizing vinculin puncta density across the three conditions. Significant differences were detected. **(C)** Bar plot showing vinculin-positive puncta size. Statistically significant differences emerged between the three experimental groups. **(D)** Box plot depicting cell stiffnesses measured on neurons grown above the three examined substrates. A significant reduction in cell stiffness was found in the case of fSLG-interfaced cells. Significance: *p < 0.05, **p < 0.01, ***p < 0.001.

To point out if the two graphene substrates may have had an impact on focal adhesions (FAs), we attempted an evaluation of their distribution and size using total internal fluorescence microscopy (TIRF). For this purpose, we performed an immunostaining assay fluorescently labeling vinculin, a cytoskeletal protein associated with the cytosolic protein complex of FAs, together with β-tubulin III (highlighting neuronal cytoskeleton), after 8–9 DIV (Figure 5A; top and bottom rows, respectively). FAs analysis was conducted visualizing vinculin-positive regions by TIRF, confining in this way the analysis to a volume extending just about a hundred of nanometers from the graphene surface (see section “Materials and Methods”). This approach made it possible to exclude from the analysis the majority of the signal coming from cytosolic vinculin not associated with FAs. β-tubulin III signal was instead acquired as a normal epi-fluorescence signal. We evaluated vinculin puncta density across the three kinds of substrates highlighting a significant reduction in puncta densities in graphene substrates (Figure 5B; 0.49 ± 0.12 puncta/μm^2^ for glass controls, *n* = 12 fields analyzed; 0.37 ± 0.05 puncta/μm^2^ for pSLG, *n* = 12; 0.31 ± 0.11 puncta/μm^2^ for fSLG, *n* = 12). Interestingly, when we estimated the average size of vinculin puncta detected across the three conditions, we observed that pSLG and fSLG induced an opposite effect when compared with glass controls. Neuronal cells interfaced with pSLG were characterized by larger puncta, while cells interfaced with fSLG presented significantly smaller ones than controls (Figure 5C; 0.157 ± 0.015 μm^2^ for glass controls, *n* = 12 fields analyzed; 0.173 ± 0.010 μm^2^ for pSLG, *n* = 12; 0.125 ± 0.011 μm^2^ for fSLG, *n* = 12). These values allow us to assume that different mechanisms were involved in FAs modulation by pSLG and fSLG.

In order to evaluate if pSLG and fSLG substrates impacted the cellular mechanoadaptation of interfaced hippocampal neurons, we have evaluated cell stiffness across the three experimental conditions. The rigidity of a cell is determined by a complex tensional equilibrium established within the cell as an adaptation to the mechanical and chemical cues provided by the extracellular environment (Azadi et al., 2019). In *in vitro* systems, this condition includes the compliance of the culturing substrate (Byfield et al., 2009; Tee et al., 2011), its chemical functionalization (Peyton et al., 2006), or micro and nano morphology (Yang et al., 2020). All these factors could induce cell stiffening or softening through an alteration in cytoskeletal structure/organization eventually mediated by a reshaping of FAs number and dimensions too (Saphirstein et al., 2013; Gupta et al., 2016). In our study, we have probed cell stiffness values of cells interfaced with pSLG, fSLG and glass control by means of AFM force-indentation experiments (Luo et al., 2016). We have described cell stiffness in terms of Young’s elastic modulus (E), a parameter commonly accepted to qualitatively quantify the rigidity of a cell (Ding et al., 2017). We discovered that pSLG did not affect neuronal cell stiffness but, conversely, fSLG interfaced cells appeared significantly softer than glass controls (Figure 5A; 6.2 ± 2.6 kPa for glass controls, *n* = 168 cells analyzed; 6.2 ± 2.8 for pSLG, *n* = 203; 4.0 ± 4.0 kPa for fSLG, *n* = 140).

## Discussion

Herein, we fabricated high-quality single-layer graphene films by CVD on flat Cu foils and subsequently transferred them on supporting glass coverslips. We chemically functionalized graphene substrates with carboxyl groups by surface insertion of phenylacetic acid moieties. Both pristine graphene films (pSLG) and functionalized ones (fSLG) were characterized by high chemical and structural quality, and demonstrated to be nanometrically flat on a length scale comparable with neuronal cells processes (about 1–5 μm). Indeed, from a surface morphology point of view, the two graphene substrates were perceived by the cells to be comparatively as even as control glass substrates (Figure 1; Rajnicek et al., 1997; Fan et al., 2002; Baranes et al., 2012).

The ability of graphene substrates to sustain proper cell adhesion and development was evaluated by interfacing them with primary cultures of rat hippocampus. We assessed the content of neuronal and glial cells above pristine and carboxyl-functionalized graphene substrates in comparison to glass controls and no significative differences emerged between the cellular networks developed above the three substrates (Figure 2). This result confirmed once again the extremely good cyto-compatibility of graphene-based materials (Lee et al., 2011; Li et al., 2011; Pampaloni et al., 2018a) and, for the first time, allowed us to state that a similar behavior is also found in the case of a covalent insertion of phenylacetic acid moieties on it.

Subsequently, we moved to a multi-technique approach, combining immunofluorescence, patch-clamping electrophysiology and scanning probe microscopy to evaluate the morphological and functional adaptation of the neuronal networks interfaced to graphene substrates for 8–9 DIV. Overall, our results demonstrate that the morphology of hippocampal cell networks developed above pSLG and fSLG substrates are almost identical to that of glass controls. Electrophysiology showed that also neuronal passive properties (R_m_ and C_m_) were not significantly different, an indication of similar plasma membrane condition and cellular dimensions (Cellot et al., 2011). Interestingly, network spontaneous activity was significantly higher in terms of frequency on pSLG-interfaced networks, but lower in fSLG-interfaced ones (Figure 3). This result highlighted an opposite modulation of neuronal cells electrical activity by the two graphene substrates, presumably indicating that two different adaptation mechanisms are involved.

In the attempt to shed some light on this aspect, we extended our investigation to the synaptic adaptation of the network by measuring the miniature PSCs of TTX-silenced networks across the three conditions. This study allowed to decouple within the network activity the contribution of dynamical components from structural components. We discovered that both graphene substrates have no effect on both the amplitude and frequency of miniature events compared to controls (Figure 4). This result suggests that the alterations in the spontaneous postsynaptic activity described earlier in graphene-interfaced neurons do not involve any structural changes in the presynaptic release probability or the number of synaptic contacts, as well as on postsynaptic receptor sensitivity (Raastad et al., 1992).

To evaluate the possibility that graphene substrates alter the network electrical activity as a side effect of a more complex, surface-mediated, tensional adaptation of the cell, we evaluated two cellular aspects connected to surface properties and mechanoadaptation: the focal adhesions organization and the cellular stiffness. In the first case, compared to controls, we highlighted a reduction in FAs densities across graphene substrates, which are particularly significative in fSLG-interfaced cells. Interestingly, FAs size was oppositely regulated by the two substrates: pSLG cells presented larger FAs while fSLG FAs were significantly smaller. This result apparently underlines for cells on pSLG an adaptive mechanism where fewer FAs are compensated by their larger size. Conversely, in fSLG-interfaced cells, this compensation is not activated and both the number and the size of FAs were reduced. Because FAs represent the fundamental cellular structure responsible for cytoskeleton-mediated cellular mechanoadaptation, we evaluated through AFM force-indentations the stiffness of neuronal cells developed above graphene substrates. We pointed out that, while the stiffness of pSLG-interfaced neurons did not change compared to control cells, fSLG cells resulted significantly softer than controls (Figure 5). Cell softening may be the eventual result of an impaired ability of the cell to adjust its internal tension through actomyosin-mediated cytoskeletal reorganization (Lessey et al., 2012) mainly due to the low density and size of the FAs that mechanically link the cell and the extracellular environment. Indeed, despite the fact further studies will be necessary to elucidate the electrophysiological impact of fSLG on neuronal firing and excitability, we have demonstrated how the physicochemical properties of a carboxyl-functionalized graphene substrate could impact not only the electrical activity of interfaced cells, but also their mechanoadaptation.

## Conclusion

This study compared the (morpho)mechanical and functional adaptation of rat primary hippocampal neurons when interfaced with surfaces covered with pristine single-layer graphene and functionalized graphene endowed with carboxylic functional groups. Our results confirmed the intrinsic ability of single-layer graphene layered on glass to boost spontaneous neuronal activity highlighting, on the other hand, the reduction in the electrical activity inducible by the -COOH functionalization. On these substrates, neurons showed a significant decrease in the frequency of spontaneous postsynaptic currents, coupled to a reduced average cell stiffness and altered focal adhesion organization in respect to controls. The reduced electrical activity observed in fSLG-interfaced neurons may be an indirect consequence of the altered mechanical properties of the cells or, instead, the result of an independent functional adaptation mechanism taking place in neurons.

Further studies will be necessary to address this open question about neuronal mechanoadaptation as well as the specific role played by different surface chemistries (e.g., amine and methyl functional groups).

Overall, we have here demonstrated that two graphene substrates, pristine and carboxylic-functionalized, could be alternatively used to intrinsically promote or depress neuronal activity in primary hippocampal cultures. We believe that neuronal implants endowed with pSLG and fSLG may be applied in future as passive neuromodulation devices potentially able to treat CNS diseases associated with a local dysregulation in neuronal activity (Edwards et al., 2012; Sullivan et al., 2021).

## Data Availability Statement

The raw data supporting the conclusions of this article will be made available by the authors, without undue reservation.

## Ethics Statement

All animal procedures were conducted following the indications of the National Institutes of Health and international and institutional standards for the care and use of animals in research. All experiments were performed in agreement with the Italian law (decree 26/14) and the European Union (EU) guidelines (2007/526/CE and 2010/63/UE) and were approved by the local authority veterinary service and by our institutional (SISSA-ISAS) ethical committee. All efforts were made to minimize animal suffering and reduce the number of animals necessary to accomplish our studies.

## Author Contributions

DS, AC, and EP-A conceived and designed the experiments. PE, FZ, HH, CW, RT, AC, GR, and EP-A performed the experiments. FZ, PE, and DS performed formal data analysis. DS, AG, and MP performed funding acquisition. AG, PG, and MP performed resources. DS, FZ, AC, and EP-A wrote the original manuscript. All authors contributed to the article and approved the submitted version.

## Conflict of Interest

The authors declare that this study received funding from AXA Foundation for Research. The funder was not involved in the study design, collection, analysis, interpretation of data, the writing of this article or the decision to submit it for publication.

## Publisher’s Note

All claims expressed in this article are solely those of the authors and do not necessarily represent those of their affiliated organizations, or those of the publisher, the editors and the reviewers. Any product that may be evaluated in this article, or claim that may be made by its manufacturer, is not guaranteed or endorsed by the publisher.

## References

[B1] Arima Y. Iwata H. (2007). Effect of wettability and surface functional groups on protein adsorption and cell adhesion using well-defined mixed self-assembled monolayers. *Biomaterials* 28 3074–3082. 10.1016/j.biomaterials.2007.03.013 17428532

[B2] Azadi S. Tafazzoli-Shadpour M. Soleimani M. Warkiani M. E. (2019). Modulating cancer cell mechanics and actin cytoskeleton structure by chemical and mechanical stimulations. *J. Biomed. Mater. Res. A* 107 1569–1581. 10.1002/jbm.a.36670 30884131

[B3] Baranes K. Chejanovsky N. Alon N. Sharoni A. Shefi O. (2012). Topographic cues of nano-scale height direct neuronal growth pattern. *Biotechnol. Bioeng.* 109 1791–1797. 10.1002/bit.24444 22252990

[B4] Bottari G. Ángeles Herranz M. Wibmer L. Volland M. Rodríguez-Pérez L. Guldi D. M. (2017). Chemical functionalization and characterization of graphene-based materials. *Chem. Soc. Rev.* 46 4464–4500. 10.1039/c7cs00229g 28702571

[B5] Byfield F. J. Reen R. K. Shentu T. P. Levitan I. Gooch K. J. (2009). Endothelial actin and cell stiffness is modulated by substrate stiffness in 2D and 3D. *J. Biomech.* 42 1114–1119. 10.1016/j.jbiomech.2009.02.012 19356760 PMC2893018

[B6] Cançado L. G. Jorio A. Ferreira E. H. M. Stavale F. Achete C. A. Capaz R. B. (2011). Quantifying defects in graphene via Raman spectroscopy at different excitation energies. *Nano Lett.* 11 3190–3196. 10.1021/nl201432g 21696186

[B7] Cellot G. Cilia E. Cipollone S. Rancic V. Sucapane A. Giordani S. (2009). Carbon nanotubes might improve neuronal performance by favouring electrical shortcuts. *Nat. Nanotechnol.* 4 126–133. 10.1038/nnano.2008.374 19197316

[B8] Cellot G. Lagonegro P. Tarabella G. Scaini D. Fabbri F. Iannotta S. (2016). PEDOT:PSS interfaces support the development of neuronal synaptic networks with reduced neuroglia response in vitro. *Front. Neurosci.* 9:521. 10.3389/fnins.2015.00521 26834546 PMC4712304

[B9] Cellot G. Toma F. M. Varley Z. K. Laishram J. Villari A. Quintana M. (2011). Carbon nanotube scaffolds tune synaptic strength in cultured neural circuits: novel frontiers in nanomaterial-tissaue interactions. *J. Neurosci.* 31 12945–12953. 10.1523/JNEUROSCI.1332-11.2011 21900573 PMC6623399

[B10] Criado A. Melchionna M. Marchesan S. Prato M. (2015). The covalent functionalization of graphene on substrates. *Angew. Chem. Int. Ed.* 54 10734–10750. 10.1002/anie.201501473 26242633

[B11] De La Rosa C. J. L. Sun J. Lindvall N. Cole M. T. Nam Y. Löffler M. (2013). Frame assisted H2O electrolysis induced H2 bubbling transfer of large area graphene grown by chemical vapor deposition on Cu. *Appl. Phys. Lett.* 102:022101. 10.1063/1.4775583

[B12] Deng Y. H. Li L. H. He J. Li M. Zhang Y. Wang X. M. (2015). Self-assembled monolayers of alkanethiolates on surface chemistry groups in osteosarcoma cells. *Mol. Med. Rep.* 11 975–981. 10.3892/mmr.2014.2876 25373556 PMC4262499

[B13] Ding Y. Xu G. K. Wang G. F. (2017). On the determination of elastic moduli of cells by AFM based indentation. *Sci. Rep.* 7:45575. 10.1038/srep45575 28368053 PMC5377332

[B14] Dong X. Wang P. Fang W. Su C. Y. Chen Y. H. Li L. J. (2011). Growth of large-sized graphene thin-films by liquid precursor-based chemical vapor deposition under atmospheric pressure. *Carbon* 49 3672–3678. 10.1016/j.carbon.2011.04.069

[B15] Edwards C. T. Zrinzo L. Limousin P. Foltynie T. (2012). Deep brain stimulation in the treatment of chorea. *Mov. Disord.* 27 357–363. 10.1002/mds.23967 21997283

[B16] Fabbro A. Scaini D. León V. Vázquez E. Cellot G. Privitera G. (2016). Graphene-based interfaces do not alter target nerve cells. *ACS Nano* 10 615–623. 10.1021/acsnano.5b05647 26700626

[B17] Fan Y. W. Cui F. Z. Hou S. P. Xu Q. Y. Chen L. N. Lee I. S. (2002). Culture of neural cells on silicon wafers with nano-scale surface topograph. *J. Neurosci. Methods* 120 17–23. 10.1016/S0165-0270(02)00181-4 12351203

[B18] Faugeras C. Amado M. Kossacki P. Orlita M. Sprinkle M. Berger C. (2009). Tuning the electron-phonon coupling in multilayer graphene with magnetic fields. *Phys. Rev. Lett.* 103 1–4. 10.1103/PhysRevLett.103.186803 19905824

[B19] Ferrari A. C. Meyer J. C. Scardaci V. Casiraghi C. Lazzeri M. Mauri F. (2006). Raman spectrum of graphene and graphene layers. *Phys. Rev. Lett.* 97 1–4. 10.1103/PhysRevLett.97.187401 17155573

[B20] Franceschi Biagioni A. Cellot G. Pati E. Lozano N. Ballesteros B. Casani R. (2021). Graphene oxide prevents lateral amygdala dysfunctional synaptic plasticity and reverts long lasting anxiety behavior in rats. *Biomaterials* 271:120749. 10.1016/j.biomaterials.2021.120749 33714913

[B21] Franco M. Alves R. Perinka N. Tubio C. Costa P. Lanceros-Mendéz S. (2020). Water-based graphene inks for all-printed temperature and deformation sensors. *ACS Appl. Electron. Mater.* 2 2857–2867. 10.1021/acsaelm.0c00508

[B22] Grimm K. B. Oberleithner H. Fels J. (2014). Fixed endothelial cells exhibit physiologically relevant nanomechanics of the cortical actin web. *Nanotechnology* 25:215101. 10.1088/0957-4484/25/21/215101 24786855

[B23] Grishagin I. V. (2015). Automatic cell counting with ImageJ. *Anal. Biochem.* 473 63–65. 10.1016/j.ab.2014.12.007 25542972

[B24] Gupta M. Doss B. Lim C. T. Voituriez R. Ladoux B. (2016). Single cell rigidity sensing: a complex relationship between focal adhesion dynamics and large-scale actin cytoskeleton remodeling. *Cell Adh. Migr.* 10 554–567. 10.1080/19336918.2016.1173800 27050660 PMC5079392

[B25] Hossain Z. Walsh M. A. Hersam M. C. (2010). Hossain_scanning tunneling microscopy, spectroscopy, and nanolithography of epitaxial graphene chemically Modified with aryl moieties. *J. Am. Chem. Soc.* 29 15399–15403. 10.1021/ja107085n 20932052

[B26] Jäkel S. Dimou L. (2017). Glial cells and their function in the adult brain: a journey through the history of their ablation. *Front. Cell. Neurosci.* 11:24. 10.3389/fncel.2017.00024 28243193 PMC5303749

[B27] Jeong J. T. Choi M. K. Sim Y. Lim J. T. Kim G. S. Seong M. J. (2016). Effect of graphene oxide ratio on the cell adhesion and growth behavior on a graphene oxide-coated silicon substrate. *Sci. Rep.* 6:33835. 10.1038/srep33835 27652886 PMC5031981

[B28] Jiang F. X. Lin D. C. Horkay F. Langrana N. A. (2011). Probing mechanical adaptation of neurite outgrowth on a hydrogel material using atomic force microscopy. *Ann. Biomed. Eng.* 39 706–713. 10.1007/s10439-010-0194-0 21063777 PMC3615638

[B29] Karki N. Tiwari H. Tewari C. Rana A. Pandey N. Basak S. (2020). Functionalized graphene oxide as a vehicle for targeted drug delivery and bioimaging applications. *J. Mater. Chem. B* 8 8116–8148. 10.1039/d0tb01149e 32966535

[B30] Keselowsky B. G. Collard D. M. García A. J. (2004). Surface chemistry modulates focal adhesion composition and signaling through changes in integrin binding. *Biomaterials* 25 5947–5954. 10.1016/j.biomaterials.2004.01.062 15183609

[B31] Kitko K. E. Hong T. Lazarenko R. M. Ying D. Xu Y. Q. Zhang Q. (2018). Membrane cholesterol mediates the cellular effects of monolayer graphene substrates. *Nat. Commun.* 9:796. 10.1038/s41467-018-03185-0 29476054 PMC5824811

[B32] Lee J. W. Serna F. Nickels J. Schmidt C. E. (2006). Carboxylic acid-functionalized conductive polypyrrole as a bioactive platform for cell adhesion. *Biomacromolecules* 7 1692–1695. 10.1021/bm060220q 16768385 PMC2548274

[B33] Lee W. C. Lim C. H. Y. X. Shi H. Tang L. A. L. Wang Y. Lim C. T. (2011). Origin of enhanced stem cell growth and differentiation on graphene and graphene oxide. *ACS Nano* 5 7334–7341. 10.1021/nn202190c 21793541

[B34] Lessey E. C. Guilluy C. Burridge K. (2012). From mechanical force to RhoA activation. *Biochemistry* 51 7420–7432. 10.1021/bi300758e 22931484 PMC3567302

[B35] Lévy R. Maaloum M. (2002). Measuring the spring constant of atomic force microscope cantilevers: thermal fluctuations and other methods. *Nanotechnology* 13 33–37. 10.1088/0957-4484/13/1/307

[B36] Li N. Zhang X. Song Q. Su R. Zhang Q. Kong T. (2011). The promotion of neurite sprouting and outgrowth of mouse hippocampal cells in culture by graphene substrates. *Biomaterials* 32 9374–9382. 10.1016/j.biomaterials.2011.08.065 21903256

[B37] Lovat V. Pantarotto D. Lagostena L. Cacciari B. Grandolfo M. Righi M. (2005). Carbon nanotube substrates boost neuronal electrical signaling. *Nano Lett.* 5 1107–1110. 10.1021/nl050637m 15943451

[B38] Lu Y. Lyu H. Richardson A. G. Lucas T. H. Kuzum D. (2016). Flexible neural electrode array based-on porous graphene for cortical microstimulation and sensing. *Sci. Rep.* 6:33526. 10.1038/srep33526 27642117 PMC5027596

[B39] Luo Q. Kuang D. Zhang B. Song G. (2016). Cell stiffness determined by atomic force microscopy and its correlation with cell motility. *Biochim. Biophys. Acta Gen. Subj.* 1860 1953–1960. 10.1016/j.bbagen.2016.06.010 27288584

[B40] Malard L. M. Pimenta M. A. Dresselhaus G. Dresselhaus M. S. (2009). Raman spectroscopy in graphene. *Phys. Rep.* 473 51–87. 10.1016/j.physrep.2009.02.003

[B41] Nayak T. R. Andersen H. Makam V. S. Khaw C. Bae S. Xu X. (2011). Graphene for controlled and accelerated osteogenic differentiation of human mesenchymal stem cells. *ACS Nano* 5 4670–4678. 10.1021/nn200500h 21528849

[B42] Nečas D. Klapetek P. (2012). Gwyddion: an open-source software for SPM data analysis. *Cent. Eur. J. Phys.* 10 181–188. 10.2478/s11534-011-0096-2

[B43] Nguyen V. T. Le H. D. Nguyen V. C. Ngo T. T. T. Le D. Q. Nguyen X. N. (2013). Synthesis of multi-layer graphene films on copper tape by atmospheric pressure chemical vapor deposition method. *Adv. Natural Sci. Nanosci. Nanotechnol.* 4:35012. 10.1088/2043-6262/4/3/035012

[B44] Pampaloni N. P. Lottner M. Giugliano M. Matruglio A. D’Amico F. Prato M. (2018a). Single-layer graphene modulates neuronal communication and augments membrane ion currents. *Nat. Nanotechnol.* 13 755–764. 10.1038/s41565-018-0163-6 29892019

[B45] Pampaloni N. P. Scaini D. Perissinotto F. Bosi S. Prato M. Ballerini L. (2018b). Sculpting neurotransmission during synaptic development by 2D nanostructured interfaces. *Nanomed. Nanotechnol. Biol. Med.* 14 2521–2532. 10.1016/j.nano.2017.01.020 28552645

[B46] Paulus G. L. C. Wang Q. H. Strano M. S. (2013). Covalent electron transfer chemistry of graphene with diazonium salts. *Acc. Chem. Res.* 46 160–170. 10.1021/ar300119z 22946516

[B47] Peyton S. R. Raub C. B. Keschrumrus V. P. Putnam A. J. (2006). The use of poly(ethylene glycol) hydrogels to investigate the impact of ECM chemistry and mechanics on smooth muscle cells. *Biomaterials* 27 4881–4893. 10.1016/j.biomaterials.2006.05.012 16762407

[B48] Raastad M. Storm J. F. Andersen P. (1992). Putative single quantum and single fibre excitatory postsynaptic currents show similar amplitude range and variability in rat hippocampal slices. *Eur. J. Neurosci.* 4 113–117. 10.1111/j.1460-9568.1992.tb00114.x 12106447

[B49] Rago I. Rauti R. Bevilacqua M. Calaresu I. Pozzato A. Cibinel M. (2019). Carbon nanotubes, directly grown on supporting surfaces, improve neuronal activity in hippocampal neuronal networks. *Adv. Biosyst.* 3 1–13. 10.1002/adbi.201800286 32627414

[B50] Rajnicek A. M. Britland S. McCaig C. D. (1997). Contact guidance of CNS neurites on grooved quartz: influence of groove dimensions, neuronal age and cell type. *J. Cell Sci.* 110 2905–2913. 10.1242/jcs.110.23.2905 9359873

[B51] Randviir E. P. Brownson D. A. C. Banks C. E. (2014). A decade of graphene research: production, applications and outlook. *Mater. Today* 17 426–432. 10.1016/j.mattod.2014.06.001

[B52] Rauti R. Secomandi N. Martín C. Bosi S. Severino F. P. U. Scaini D. (2020b). Tuning neuronal circuit formation in 3D polymeric scaffolds by introducing graphene at the bio/material interface. *Adv. Biosyst.* 4 1–12. 10.1002/adbi.201900233 32293163

[B53] Rauti R. Cellot G. D’Andrea P. Colliva A. Scaini D. Tongiorgi E. (2020a). BDNF impact on synaptic dynamics: extra or intracellular long-term release differently regulates cultured hippocampal synapses. *Mol. Brain* 13 1–16. 10.1186/s13041-020-00582-9 32183860 PMC7079446

[B54] Reina A. Jia X. Ho J. Nezich D. Son H. Bulovic V. (2009). Large area, few-layer graphene films on arbitrary substrates by chemical vapor deposition. *Nano Lett.* 9 30–35. 10.1021/nl801827v 19046078

[B55] Reina G. González-Domínguez J. M. Criado A. Vázquez E. Bianco A. Prato M. (2017). Promises, facts and challenges for graphene in biomedical applications. *Chem. Soc. Rev.* 46 4400–4416. 10.1039/c7cs00363c 28722038

[B56] Ren Y. J. Zhang H. Huang H. Wang X. M. Zhou Z. Y. Cui F. Z. (2009). *In vitro* behavior of neural stem cells in response to different chemical functional groups. *Biomaterials* 30 1036–1044. 10.1016/j.biomaterials.2008.10.028 19026444

[B57] Ryoo S. Kim Y. Kim M. Min D. (2010). Behaviors of NIH-3T3 Fibroblasts on. *ACS Nano* 4 6587–6598. 20979372 10.1021/nn1018279

[B58] Saphirstein R. J. Gao Y. Z. Jensen M. H. Gallant C. M. Vetterkind S. Moore J. R. (2013). the focal adhesion: a regulated component of aortic stiffness. *PLoS One* 8:e62461. 10.1371/journal.pone.0062461 23626821 PMC3633884

[B59] Sarnat H. B. Nochlin D. Born D. E. (1998). Neuronal nuclear antigen (NeuN): a marker of neuronal maturation in the early human fetal nervous system. *Brain Dev.* 20 88–94. 10.1016/S0387-7604(97)00111-3 9545178

[B60] Schindelin J. Arganda-Carreras I. Frise E. Kaynig V. Longair M. Pietzsch T. (2012). Fiji: an open-source platform for biological-image analysis. *Nat. Methods* 9 676–682. 10.1038/nmeth.2019 22743772 PMC3855844

[B61] Shin S. R. Li Y. C. Jang H. L. Khoshakhlagh P. Akbari M. Nasajpour A. (2016). Graphene-based materials for tissue engineering. *Adv. Drug Deliv. Rev.* 105 255–274. 10.1016/j.addr.2016.03.007 27037064 PMC5039063

[B62] Sneddon I. N. (1965). The relation between load and penetration in the axisymmetric boussinesq problem for a punch of arbitrary profile. *Int. J. Eng. Sci.* 3 47–57. 10.1016/0020-7225(65)90019-4

[B63] Sullivan C. R. P. Olsen S. Widge A. S. (2021). Deep brain stimulation for psychiatric disorders: from focal brain targets to cognitive networks. *Neuroimage* 225:117515. 10.1016/j.neuroimage.2020.117515 33137473 PMC7802517

[B64] Tang M. Song Q. Li N. Jiang Z. Huang R. Cheng G. (2013). Enhancement of electrical signaling in neural networks on graphene films. *Biomaterials* 34 6402–6411. 10.1016/j.biomaterials.2013.05.024 23755830

[B65] Tee S. Y. Fu J. Chen C. S. Janmey P. A. (2011). Cell shape and substrate rigidity both regulate cell stiffness. *Biophys. J.* 100 L25–L27. 10.1016/j.bpj.2010.12.3744 21354386 PMC3043219

[B66] Torrisi F. Hasan T. Wu W. Sun Z. Lombardo A. Kulmala T. S. (2012). Inkjet-printed graphene electronics. *ACS Nano* 6 2992–3006. 10.1021/nn2044609 22449258

[B67] Ulloa L. S. Perissinotto F. Rago I. Goldoni A. Santoro R. Pesce M. (2021). Carbon nanotubes substrates alleviate pro-calcific evolution in porcine valve interstitial cells. *Nanomaterials (Basel)* 11:2724. 10.3390/nano11102724 34685165 PMC8538037

[B68] Xu M. Zhu J. Wang F. Xiong Y. Wu Y. Wang Q. (2016). Improved *in vitro* and *in vivo* biocompatibility of graphene oxide through surface modification: poly(acrylic acid)-functionalization is superior to PEGylation. *ACS Nano* 10 3267–3281. 10.1021/acsnano.6b00539 26855010

[B69] Yang L. Gao Q. Ge L. Zhou Q. Warszawik E. M. Bron R. (2020). Topography induced stiffness alteration of stem cells influences osteogenic differentiation. *Biomater. Sci.* 8 2638–2652. 10.1039/d0bm00264j 32248219

[B70] Zhang L. Lu Z. Zhao Q. Huang J. Shen H. Zhang Z. (2011). Enhanced chemotherapy efficacy by sequential delivery of siRNA and anticancer drugs using PEI-grafted graphene oxide. *Small* 7 460–464. 10.1002/smll.201001522 21360803

[B71] Zhou K. Motamed S. Thouas G. A. Bernard C. C. Li D. Parkington H. C. (2016). Graphene functionalized scaffolds reduce the inflammatory response and supports endogenous neuroblast migration when implanted in the Adult Brain. *PLoS One* 11:e0151589. 10.1371/journal.pone.0151589 26978268 PMC4792446

[B72] Zhou X. Wang F. Zhu Y. Liu Z. (2011). Graphene modified LiFePO4 cathode materials for high power lithium ion batteries. *J. Mater. Chem.* 21 3353–3358. 10.1039/c0jm03287e

