# Supplementary material for: Bidirectional Modulation of Neuronal Cells Electrical and Mechanical Properties Through Pristine and Functionalized Graphene Substrates
Source: Front Neurosci. 2022 Jan 11;15:811348. doi: 10.3389/fnins.2021.811348 (PMC8788235; doi:10.3389/fnins.2021.811348)
Supplement: Supplementary file 1 [file Table_1.pdf]

Table S1

Sample	I_D/I_G		$\Delta I_D/I_G$	nD (cm^{-2})
	pSLG	fSLG		
1	0.04	0.33	0.29	6.52×10^{10}
2	0.05	0.23	0.18	4.04×10^{10}
3	0.03	0.19	0.16	3.60×10^{10}
4	0.03	0.23	0.20	4.49×10^{10}
5	0.08	0.15	0.07	1.57×10^{10}
6	0.05	0.32	0.27	6.07×10^{10}

Table S1. *The table summarizes the I_D/I_G ratio for pSLG and fSLG, and defect density for fSLG. Values are relative to six graphene samples and represent measurements performed before the chemical functionalization (pSLG, grey column) and after (fSLG, red column).*
